# A case-control study of breast cancer risk factors in 7,663 women in Malaysia

**DOI:** 10.1371/journal.pone.0203469

**Published:** 2018-09-14

**Authors:** Min-Min Tan, Weang-Kee Ho, Sook-Yee Yoon, Shivaani Mariapun, Siti Norhidayu Hasan, Daphne Shin-Chi Lee, Tiara Hassan, Sheau-Yee Lee, Sze-Yee Phuah, Kavitta Sivanandan, Patsy Pei-Sze Ng, Nadia Rajaram, Maheswari Jaganathan, Suniza Jamaris, Tania Islam, Kartini Rahmat, Farhana Fadzli, Anushya Vijayananthan, Pathmanathan Rajadurai, Mee-Hong See, Meow-Keong Thong, Nur Aishah Mohd Taib, Cheng-Har Yip, Soo-Hwang Teo

**Affiliations:** 1 Department of Applied Mathematics, Faculty of Engineering, University of Nottingham Malaysia Campus, Semenyih, Selangor, Malaysia; 2 Cancer Research Malaysia, Subang Jaya, Selangor, Malaysia; 3 Department of Surgery, Faculty of Medicine, University of Malaya, Kuala Lumpur, Malaysia; 4 Biomedical Imaging Department, Faculty of Medicine, University of Malaya, Kuala Lumpur, Malaysia; 5 Sime Darby Medical Centre, Subang Jaya, Selangor, Malaysia; 6 Jeffrey Cheah School of Medicine and Health Sciences, Monash University Malaysia Campus, Subang Jaya, Selangor, Malaysia; 7 Department of Paediatrics, Faculty of Medicine, University of Malaya, Kuala Lumpur, Malaysia; University of Oklahoma Health Sciences Center, UNITED STATES

## Abstract

**Background:**

Breast cancer risk factors have been examined extensively in Western setting and more developed Asian cities/countries. However, there are limited data on developing Asian countries. The purpose of this study was to examine breast cancer risk factors and the change of selected risk factors across birth cohorts in Malaysian women.

**Methods:**

An unmatched hospital based case-control study was conducted from October 2002 to December 2016 in Selangor, Malaysia. A total of 3,683 cases and 3,980 controls were included in this study. Unconditional logistic regressions, adjusted for potential confounding factors, were conducted. The breast cancer risk factors were compared across four birth cohorts by ethnicity.

**Results:**

Ever breastfed, longer breastfeeding duration, a higher soymilk and soy product intake, and a higher level of physical activity were associated with lower risk of breast cancer. Chinese had the lowest breastfeeding rate, shortest breastfeeding duration, lowest parity and highest age of first full term pregnancy.

**Conclusions:**

Our study shows that breastfeeding, soy intake and physical activity are modifiable risk factors for breast cancer. With the increasing incidence of breast cancer there is an urgent need to educate the women about lifestyle intervention they can take to reduce their breast cancer risk.

## Background

Breast cancer risk factors have been examined extensively and the common ones include early age of menarche, late age of menopause, short breastfeeding duration, late age of first full term pregnancy, nulliparity and low parity [1–5]. However, most of these studies were conducted predominantly in developed countries in a Western setting. Although a limited number of studies examining women living in Asian countries also supported the association of these common risk factors with breast cancer [6–10], they were conducted in the more developed Asian cities/countries, or have been limited to sample sizes of several hundred women and mostly limited to one ethnicity. Therefore, there is a need to conduct a more extensive study with a larger sample size to determine whether these risk factors also play a similar role among Asian populations in developing countries, as this evidence should contribute importantly to the development of appropriate strategies for breast cancer prevention and control in Asia.

Malaysia offers a unique opportunity to examine breast cancer risk factors in Asian populations because of its multi-cultural and multi-religious setting, both of which might influence lifestyle and reproductive characteristics, and hence, breast cancer risk. Notably, the three main ethnicities in Malaysia, namely, Malay, Chinese and Indian, represent the three largest ethnic groups in Asia. Breast cancer is the most common cancer among Malaysian women and accounted for 31% of total female cancers [11]. The age-adjusted breast cancer incidence in Malaysia is 47.4/100,000, about half of that in North America [12]. Chinese have the highest incidence (59.9/100,000) followed by Indians (54.2/100,000) and Malays (34.9/100,000) [11]. Like many developing Asian countries, Malaysia is undergoing a transition toward a Westernized diet that is high in fat and sugar, an increasingly sedentary lifestyle [13] and also experiencing changes in reproductive characteristics [14]. Thus, there is an urgent need to examine the impact of these changes on breast cancer risk.

In this paper, we report the association between clinical, exogenous hormonal, menstrual, reproductive, anthropometric and lifestyle factors with breast cancer from a hospital-based case-control study of 7,663 women in Malaysia. We also present the change of selected breast cancer-related factors across birth cohorts and their implication for breast cancer in Malaysia and potentially other developing Southeast Asian countries.

## Materials and methods

The study was approved by the Independent Ethics Committee, Ramsay Sime Darby Health Care (reference nos: 201109.4 and 201208.1), and the Medical Ethics Committee, University Malaya Medical Centre (reference no: 842.9). All participants provided written informed consent. The study was performed in accordance with the Declaration of Helsinki.

The Malaysian Breast Cancer Genetic Study (MyBrCa), initiated in 2002, is a hospital-based case-control study of breast cancer risk factors. The study participants are recruited from two participating hospitals in Selangor, Malaysia: University Malaya Medical Centre (UMMC), a public hospital, and Subang Jaya Medical Centre (SJMC), a private hospital. All patients diagnosed clinically with breast carcinoma are eligible for inclusion as cases. Cases from UMMC were recruited since October 2002, and from SJMC, since September 2012. Controls are healthy women between ages 40 and 74 with no personal history of breast cancer and recruited in the Malaysian Mammography Study (MyMammo) at UMMC and SJMC. At SJMC, MyMammo is a subsidized opportunistic mammogram screening programme that was initiated in 2011; while at UMMC, MyMammo started recruitment in 2014 from patients attending routine opportunistic screening in UMMC.

All participants were interviewed by trained interviewers at the hospitals. The participants completed questionnaire that included items related to demographics, personal and family history of cancers, history of breast surgery, menstrual and reproductive history, use of oral contraceptive and hormone replacement therapy (HRT), breast cancer diagnosis (cases only) and history of and motivation of attending mammography screening (controls) only. The participants provided a blood sample that was processed and stored.

### Statistical analysis

To date, a total of 4,056 cases and 4,145 controls were recruited and interviewed. Only participants recruited before 1 January 2017 were included in this study. After removing duplicates, males and non-breast cancer cases, the remaining cohort consists of 3,683 cases and 3,980 controls.

Ever had breast surgery was defined as whether the participant had surgery for a benign lump or cyst in the breast. Women who had sisters/mothers/daughters with breast cancer were categorized as having a first-degree family history of breast cancer. Ever used oral contraceptives and HRT was defined as at least one month of usage. Post-menopausal status was defined as no menses for the past one year. The participants were categorized as parous if they had at least one full term pregnancy (live or still birth). BMI was calculated as dividing weight (kg) by the square of height (m). Soy products intake included the consumption of tofu, fermented soybeans, tofu pudding, and soy products other than soymilk. The participants reported their average duration of strenuous, moderate and gentle physical activity of three periods: childhood (before 18 years old), young adulthood (18–30 years), and the recent years. Weekly metabolic equivalent (MET)-hours were obtained by multiplying the corresponding MET value of each intensity of physical activity (7, 4, 3 for strenuous, moderate and gentle activities, respectively) with the average time spent on physical activity [15].

Cases and controls were compared using chi-square tests for categorical variables and t-tests for continuous variables. Unconditional logistic regressions were conducted to assess the association between risk factors and breast cancer, adjusting for potential confounders and other risk factors. The first models were adjusted for age, ethnicity, and hospital; for history of breast surgery, and anthropometric and lifestyle variables, the models were adjusted for age, ethnicity and education, and only participants from private hospital were included. In the second models, other breast cancer risk factors such as age of menarche, age of menopause, ever had full term pregnancy, first degree family history of breast cancer, and age of first full term pregnancy were added when appropriate. Conditional logistic regression using hospital-, ethnicity- and age- (±5 years) matched samples and unconditional logistic regressions stratified by pre- and post-menopausal status were also conducted. However, the results were similar to the unconditional and unstratified analysis thus they are not reported here.

The participants were categorized based on their year of birth into four birth cohorts: those born before 1949, between 1950–59, between 1960–69, and after 1969, and their breast cancer risk factors were compared across the birth cohorts. To compare across ethnicity, analysis of variances (ANOVAs) were conducted for continuous variables while chi-square tests were conducted for categorical variables. To determine whether there was a change of trend in the selected variables across birth cohorts, trend analyses were conducted by entering the birth cohort variable as continuous parameter in the regression models.

All analyses were conducted using R [16].

## Results

Table 1 is the demographic comparisons of cases and controls. Controls were significantly older than cases, with mean ages of 54.0 years and 50.8 years, respectively (p<0.001) and significantly more controls had received secondary education. There were significantly more Chinese among the cases.

**Table 1:** Socio-demographic characteristics of 3,683 breast cancer cases and 3,980 controls in a hospital-based case-control study of breast cancer.

Variables	Cases (N = 3,683)		Controls (N = 3,980)		p
	N	%	N	%	
**Age (years)**					<0.001
<45	1051	29.35	595	15.02	
45–54	1268	35.41	1556	39.27	
55–64	851	23.76	1297	32.74	
>65	411	11.48	514	12.97	
**Ethnicity**					<0.001
Chinese	2481	68.74	2106	53.11	
Indian	436	12.08	860	21.69	
Malay	610	16.9	846	21.34	
Other	82	2.27	153	3.86	
**Education**					<0.001
Primary or less	214	17.16	308	8.31	
Secondary	556	44.59	2010	54.25	
Tertiary	477	38.25	1387	37.44	
**Monthly household income (RM)**					0.672
<5,000	703	57.67	2267	58.81	
5–10,000	319	26.17	1003	26.02	
>10,000	197	16.16	585	15.18	

We conducted unconditional logistic regression to examine the association of clinical, exogenous hormonal, menstrual and reproductive factors with breast cancer (Table 2). Compared with those who had never had breast surgery, participants who had breast surgery to remove cysts and lumps were 2.3 times (95% CI = 1.82–2.83) more likely to develop breast cancer after adjusting for demographics and other risk factors. First-degree family history of breast cancer was associated with 19% increased risk of breast cancer after adjusting for demographics and other risk factors. Post-menopausal women had a 52% increased risk of breast cancer after adjusting for demographics and other risk factors. The use of oral contraceptives and HRT were not significantly associated with breast cancer risk after adjustment of other breast cancer risk factors.

**Table 2:** Clinical, exogenous hormonal, and menstrual and reproductive factors and their association with breast cancer.

Variables	Cases/Controls	OR(95%CI)^3^	OR(95%CI)^4^
**Ever had breast surgery**			
No	972/1,807		
Yes	249/214	2.42(1.96–3.00)***	2.27(1.82–2.83)***
**First degree family history of breast cancer**			
No	3,170/3,478		
Yes	513/502	1.10(0.96–1.27)	1.19(1.02–1.38)*
**Ever use oral contraceptives**			
Never	2,452/2,806		
Ever	935/1,145	1.02(0.91–1.14)	0.99(0.88–1.11)
**Ever use hormonal replacement therapy**^1^			
Never	419/1,452		
Ever	51/259	0.52(0.44–0.61)***	0.48(0.4–0.58)***
**Age of menarche**			
≤12	1,178/1,611		
>12	1,714/2,329	1.04(0.94–1.15)	1.04(0.94–1.16)
**Menopausal status**			
Pre/Peri-menopausal	1,149/1,550		
Post-menopausal	1771/2,408	1.53(1.33–1.76)***	1.52(1.32–1.75)***
**Age of menopause (years)**^1^			
≤50	217/862		
>50	246/822	0.86(0.71–1.03)	0.89(0.73–1.08)
**Ever had full term pregnancy**			
Nulliparous	473/552		
Parous	2,778/3,366	1.05(0.91–1.20)	1.13(0.97–1.31)
**Parity**^2^			
1	319/337		
2	828/974	0.97(0.81–1.18)	1.09(0.82–1.46)
3	800/1,020	0.97(0.8–1.17)	0.98(0.73–1.32)
4	460/586	1.12(0.91–1.38)	0.99(0.72–1.38)
≥5	370/449	1.49(1.19–1.86)***	1.20(0.85–1.69)
**Age of first full term pregnancy**^2^			
<25	925/940		
25–29	1,039/1,506	1.09(0.87–1.37)	1.0(0.79–1.28)
≥30	744/845	1.38(1.08–1.77)*	1.29(0.99–1.67)
**Ever breastfed**^2^			
Never	532/684		
Ever	942/2,514	0.56(0.48–0.65)***	0.65(0.55–0.78)***
**Breastfeeding duration in months**^2^			
None	532/684		
≤12	766/1,678	0.60(0.51–0.7)***	0.73(0.61–0.88)***
>12	173/836	0.40(0.32–0.5)***	0.30(0.22–0.4)***

*p<0.05;

***p<0.001

^1^Natural menopause;

^2^Parous women only

^3^ Adjusted for age, ethnicity, and hospital, except history of breast surgery, which is adjusted for age, ethnicity, and education and only participants from private hospital are included

^4^Adjusted for age, ethnicity, hospital and breast cancer risk factors (age of menarche, menopausal status, ever had full term pregnancy, first degree family history of breast cancer, parity, age of first full term pregnancy, ever breastfed, breastfeeding duration when appropriate), except history of breast surgery, which is adjusted for age, ethnicity, education and relevant breast cancer risk factors, and only participants from private hospital are included

Of the menstrual and reproductive factors examined, breastfeeding had the strongest protective effect against breast cancer (Table 2). Among parous women, those who ever breastfed had 35% lower risk in the fully adjusted models; compared with those who did not breastfeed, the reduction of risk for those who breastfed between 1–12 months and those who breastfed more than 12 months was 30% and 70% respectively.

We also examined the association between anthropometric and lifestyle factors and breast cancer (Table 3). A higher BMI was associated with a lower risk of breast cancer; those who are overweight (BMI = 23.0–27.4kg/m^2^) had 33% reduced risk and those who are obese (BMI ≥ 27.5kg/m^2^) had 53% reduced risk, after controlling for other risk factors (Table 3). Those who consumed one cup or more soymilk per week and soy products once or more per week had 75% and 60% reduction in breast cancer risk, respectively. We did not find any significant association between smoking status and breast cancer. Women who drink less than 1 glass of alcohol per week and 1 glass per week or more had 55% and 48% reduced risk of breast cancer. It is noteworthy that the prevalence of those who reported alcohol intake in our cohort is low at 14%. A higher level of physical activity during childhood, young adulthood and recent period were also significantly associated with reduced risk of breast cancer before and after adjusting for other risk factors.

**Table 3:** Anthropometric and lifestyle factors and their association with breast cancer.

Variables	Cases/Controls	OR(95%CI)^1^	OR(95%CI)^2^
**Height**			
<1.53	224/399		
1.53–1.57	401/666	1.10(0.88–1.37)	1.07(0.84–1.35)
>1.57	589/950	1.08(0.88–1.34)	1.06(0.84–1.33)
**BMI**			
<23.0	654/757		
23.0–27.4	388/758	0.68(0.57–0.81)***	0.67(0.56–0.81)***
≥27.5	165/493	0.53(0.43–0.67)***	0.47(0.37–0.61)***
**Ever smoked**			
Never	1202/3,745		
Ever	93/214	0.97(0.74–1.28)	0.75(0.56–1.01)
**Alcohol intake**			
Non-drinker	1073/3,394		
Less than 1 glass per week	98/279	0.71(0.55–0.91)**	0.45(0.34–0.59)***
1 glass per week or more	71/169	0.75(0.55–1.02)	0.52(0.37–0.71)***
**Soy milk intake**			
None	795/2362		
1 cup per week or less	382/1036	1.17(1–1.37)*	1.24(1.03–1.49)*
1 cup or more per week	72/521	0.36(0.27–0.48)***	0.25(0.18–0.33)***
**Soy products intake**			
Once a week/less	403/401		
Once a week or more	614/1,476	0.39(0.33–0.47)***	0.40(0.33–0.48)***
**Physical activity, childhood (METS-hours/week)**			
<10	368/404		
10–20	282/576	0.58(0.47–0.72)***	0.55(0.44–0.7)***
>20	425/942	0.59(0.48–0.72)***	0.58(0.47–0.72)***
**Physical activity, mid-adulthood (METS-hours/week)**			
<10	524/518		
10–20	307/639	0.52(0.43–0.63)***	0.50(0.41–0.61)***
>20	243/785	0.35(0.29–0.43)***	0.35(0.29–0.44)***
**Physical activity, recent (METS-hours/week)**			
<10	518/706		
10–20	407/759	0.73(0.61–0.87)***	0.72(0.59–0.87)***
>20	168/490	0.45(0.36–0.56)***	0.42(0.33–0.53)***

*p<0.05;

**p<0.01;

***p<0.001

^1^Adjusted for age, ethnicity, and education. Only participants from private hospital were included

^2^Adjusted for age, ethnicity, education, and breast cancer risk factors (age of menarche, menopausal status, ever had full term pregnancy, first degree family history of breast cancer, parity, age of first full term pregnancy, ever breastfed, breastfeeding duration, ever had breast surgery when appropriate). Only participants from private hospital were included

### Change of risk factors by birth cohorts

**Fig 1:**
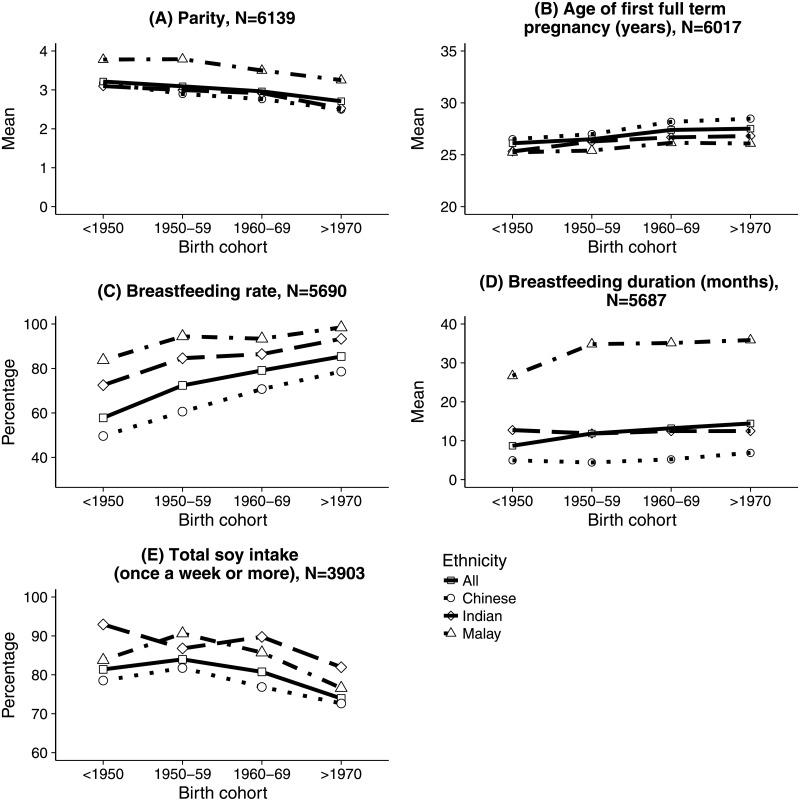
Change of breast cancer risk factors across birth cohorts.

We examined the change of all risk factors across birth cohorts of both controls and cases in the three major ethnic groups in Malaysia and here we report the variables that had significantly changed across birth cohorts. Fig 1 showed the change of parity, age of first full term pregnancy, breastfeeding rate, breastfeeding duration and total soy intake. Compared with Indians and Malays, Chinese have the lowest parity, oldest age of first full term pregnancy, lowest breast feeding rate and shortest breastfeeding duration (p<0.001). All ethnic groups were experiencing significant reduction in parity (p<0.001 for all races) and significant increase of age of first full term pregnancy (p<0.001 for Chinese and p<0.001 for Malays and Indians) across birth cohorts.

All ethnic groups had significant increase of breastfeeding rate across birth cohorts (p<0.001). The increase was more noticeable among Chinese; there was an increase from 50% among the oldest cohort to 79% among the youngest cohort. Only Chinese had a significant increase of breastfeeding duration across birth cohorts (p<0.001); however, breastfeeding duration among Chinese remained low compared with Malays and Indians.

There was a significant decrease of total soy intake among Chinese (p<0.001) and Malay (p<0.05) across birth cohorts. Compared with Malays and Indians, Chinese consumed significantly less soy products (p<0.001). However, the number of Malays and Indians who reported their intake of soy products were small compared with that of Chinese.

## Discussion

In this hospital-based case-control study of 7,663 Malaysian women, we showed that a higher breastfeeding rate and duration, soy intake and level of physical activity were associated with a reduced risk of breast cancer among Southeast Asian women. Although Southeast Asian countries are experiencing a substantial increase in the burden of breast cancer, there have been limited studies in risk factors for breast cancer in these populations. Before the Malaysian Breast Cancer Genetic study, the previous largest study on breast cancer risk factors in Southeast Asia was from Indonesia and included 526 cases and 1,052 controls [17, 18]. A large scale prospective cohort study that followed 35,303 women in which 629 developed breast cancer has been conducted in Singapore, however, it focused mainly on soy intake and breast cancer risk and was limited to Chinese only [19]. Our current study included a large sample size and examined a wide range of breast cancer risk factors.

The strongest predictor of breast cancer in our study was breastfeeding, and the inverse association between breastfeeding and breast cancer risk is well documented [6, 7, 20–22]. Our study also showed an increasing trend of breastfeeding across birth cohorts in all ethnicity; however, among Chinese the breastfeeding rate and duration were still relatively low. The low breastfeeding rate and short breastfeeding duration may contribute to the highest breast cancer incidence (59.9/100,000) among Chinese in Malaysia compared with Indians (54.2/100,000) and Malays (34.9/100,000) [11]. Thus, the results of our study could be helpful in public health strategies to reduce risk of breast cancer through modifiable lifestyle choices including breastfeeding.

Our study also found that higher intake of soymilk and soy products is associated with lower risk of breast cancer. Soy is a major food in many parts of Asia and retrospective cross-sectional cohort studies in China and Japan show that increased soy protein intake is associated with reduced breast cancer risk in pre- and post-menopausal women [23, 24]. A study in Singapore showed that increased soy intake was significantly associated with reduced breast cancer risk among pre-menopausal women but not post-menopausal women [9] while another study in China found no significant association between soy protein intake and breast cancer risk. While there is some heterogeneity across these Asian studies, meta-analyses of observational studies in both Caucasian and Asian countries have consistently shown that high soy intake is associated to a lower risk of breast cancer, particularly among Asian women [25–30]. Given that our results shows declining soy intake across birth cohorts, future studies are required to confirm the benefit of soy in reducing population risk of breast cancer, as well as to also identify effective strategies to increase soy intake among Asian women, for whom a soy intervention may be an affordable and acceptable strategy for breast cancer prevention.

Another lifestyle factor that is shown to be associated with decreased breast cancer risk in our study is physical activity. This is consistent with the latest World Cancer Research Fund report which showed strong probable evidence that regular physical activity of various intensity decreases the risk of breast cancer among post-menopausal women while among pre-menopausal women, regular vigorous physical activity is associated with reduced risk [31]. A recent systematic review evaluated 80 studies and found that moderate-vigorous physical activity is associated with lower breast cancer risk among pre-menopausal (RR = 0.80, 95% CI = 0.74–0.87) and post-menopausal cohort studies (RR = 0.79, 95% CI = 0.76–0.84) [32]. Another systematic review that examined the dose-response between physical activity and major non-communicable diseases, which included breast cancer, found that compared with insufficiently active women, the reduction of risk of breast cancer among the low active, moderately active and highly active was 3%, 6% and 14% respectively [33]. Compared with other populations, Malaysian women have a higher prevalence of physical inactivity [34] and in our study there was no significant change of physical activity across birth cohorts. Thus, there is a need to construct innovative strategy to increase the level of physical activity in order to reduce future breast cancer risk.

In our study, two risk factors were associated with breast cancer risk in the contradictory direction. First, the consumption of alcohol was associated with a decreased risk of breast cancer in our study. The association of alcohol consumption with increased breast cancer risk has long been established [35]. However, in our study, only 6% reported an intake of more than 1 glass of alcohol per week, which is low compared with other populations. The second risk factor that had a contradictory association with breast cancer in our study was a higher BMI, which was associated with a lower risk of breast cancer after adjustment for major breast cancer risk factors. Past studies have shown that a higher BMI is associated with increased risk among post-menopausal women and reduced risk among pre-menopausal women [36]. However, when stratified by menopausal status, our analysis showed that a higher BMI was still significantly associated with lower breast cancer risk in both pre- and post-menopausal women. More studies need to be conducted among the Malaysian women to further explore the link between BMI and breast cancer risk.

In addition, our study did not find a significant association between parity, age of first full term pregnancy, age of menarche and menopause and breast cancer, which is inconsistent with other studies [2–5, 8, 10, 37–41]. Our study also found only a slight association between first-degree family history of breast cancer and increased risk of breast cancer risk, while other studies show that family history is strongly associated with increased breast cancer risk [20, 21, 39, 41–44].

Since this is a hospital-based case-control study rather than population-based, it might be subject to selection bias. The two hospitals where our participants were recruited were located in urban areas and rural Malaysian women were not included. However, it is noteworthy that these hospitals treat more than 10% of the breast cancer cases in Malaysia. The controls of our study were enriched for women who had a family history of breast cancer because they were participants of an opportunistic mammography screening programme.

In conclusion, our study shows that breastfeeding, soy intake and physical activity are modifiable risk factors for breast cancer; and with the increasing incidence of breast cancer there is an urgent need to educate the women about lifestyle intervention they can take to reduce their risk of breast cancer.

## Supporting information

S1 File Survey questions. This file contains the questionnaire items used in this study.
(DOCX)
