# Supplementary material for: A case-control study of breast cancer risk factors in 7,663 women in Malaysia
Source: PLoS One. 2018 Sep 14;13(9):e0203469. doi: 10.1371/journal.pone.0203469 (PMC6138391; doi:10.1371/journal.pone.0203469)
Supplement: S1 File — This file contains the questionnaire items used in this study. (DOCX) [file pone.0203469.s001.docx]

**Malaysia Breast Cancer Genetic Study**

1. What is your **full** name? ............................................................................

What was your maiden name (*if applicable*) ............................................................................

2. Gender  Male  Female

3. Marital Status  Single  Married  other, please specify

4. What is your IC number?

5. What is your address? ............................................................................

………………………………………………………………………...

6. What is your telephone number? -

What is your mobile phone number? -

7. What is your date of birth (dd/mm/yyyy)? //

8. What is your height (in cm or in feet and inches)? . cm **OR**  ft  in

9. What is your current weight (in kg or in pounds)? . kg **OR** . lb

10. How would you describe your ethnic origin?

Malay  Chinese  Indian  other, please specify

11. What is your highest education level?

Primary  Secondary  University  other, please specify

12. What is your average monthly household income?

<5,000  5-10,000  10-50,000  >50,000

**INFORMATION ABOUT YOUR HEALTH**

13. How much physical activity/sports do you practice?

*(please tick ✓ all boxes that apply under strenuous, moderate and gentle exercises)*

**Strenuous Exercise:**

Which normally makes your heart beats rapidly AND leaves you breathless, e.g. jogging, vigorous swimming or cycling, aerobics

**Moderate Exercise:**

Which normally leaves you exhausted but not breathless, e.g. brisk walking, dancing, easy swimming or cycling, badminton, sailing, yoga, pilates, taiqi. qigong

**Gentle Exercise:**

Which normally leaves you tired but not exhausted, e.g. walking, driving, housework (including

washing windows and polishing), gardening, DIY, golf.

| 14. | Childhood (before 18-years of age)   \| **Strenuous Exercise** \| **Moderate Exercise** \| **Gentle Exercise** \| \| --- \| --- \| --- \| \| Never  Less than 1 hour per week  1-2 hour per week  More than 2 hours per week \| Never  Less than 1 hour per week  1-2 hour per week  More than 2 hours per week \| Never  Less than 1 hour per week  1-2 hour per week  More than 2 hours per week \| |
| --- | --- | --- | --- | --- | --- | --- | --- |
| 15. | 18-30-years of age   \| **Strenuous Exercise** \| **Moderate Exercise** \| **Gentle Exercise** \| \| --- \| --- \| --- \| \| Never  Less than 1 hour per week  1-2 hour per week  More than 2 hours per week \| Never  Less than 1 hour per week  1-2 hour per week  More than 2 hours per week \| Never  Less than 1 hour per week  1-2 hour per week  More than 2 hours per week \| |
| 16. | The most recent years   \| **Strenuous Exercise** \| **Moderate Exercise** \| **Gentle Exercise** \| \| --- \| --- \| --- \| \| Never  Less than 1 hour per week  1-2 hour per week  More than 2 hours per week \| Never  Less than 1 hour per week  1-2 hour per week  More than 2 hours per week \| Never  Less than 1 hour per week  1-2 hour per week  More than 2 hours per week \| |

**INFORMATION ABOUT YOUR HEALTH**

17. Have you ever smoked cigarettes?  Yes  No

**If yes**, do you still smoke?  Yes  No

For how many years have you ever smoked?  Years

18. Have you ever drunk alcohol more than once a month on average?  Yes  No

(Alcohol includes beer, wine, hard liquor)

**If yes**, how much do you drink on average?

1 glass per day  1 glass per week  1 glass per month  Other, please specify

19. Do you drink **soya** **bean** milk regularly?  Yes  No

**If yes**, how much do you drink on average?

1 cup per week/ less  1 cup per day  1-5 cups per day  > 5 cups per day

Do you eat soy products (tofu, tofufah) regularly?

Every meal  Every day  Once a week  Other, please specify

I**NFORMATION ABOUT MENSTRUATION, PREGNANCY AND CHILDBIRTH**

20. How old were you when your periods began?  Years

21. Are you still having periods?  Yes  No

**If yes**, are they regular or irregular?  Regular  Irregular

How many days apart are your menstrual periods?  28 29  30  31 32  other, please specify: ……..

22. If you are no longer having periods, how old were you when they stopped completely?

Years

**And**, why and when (dd/mm/yyyy) did they stop?

It stopped by itself //

Uterus was removed //

Ovaries were removed //

Medication/Chemotherapy //

Other reason …………………………………………………………………………

23. Have you ever been pregnant?  Yes  No

| If Yes, please state all pregnancies (including miscarriages and still births), gender, birth year and birth weight for all children you gave birth to and number of months you breast fed | | | | | |
| --- | --- | --- | --- | --- | --- |
| Pregnancy | Child/Stillborn/  Miscarriage | Gender  (M/F) | Birthyear | Birthweight (g) | Breastfeeding  (months) |
| 1 |  |  |  |  |  |
| 2 |  |  |  |  |  |
| 3 |  |  |  |  |  |
| 4 |  |  |  |  |  |
| 5 |  |  |  |  |  |
| 6 |  |  |  |  |  |
| 7 |  |  |  |  |  |
| 8 |  |  |  |  |  |

24. Have you ever used the contraceptive pill?  Yes  No

**If yes**, for how long, in total? ……………………………………………………..

**And** are you currently taking the contraceptive pill?  Yes  No

25. Have you ever used hormone replacement therapy (HRT)?  Yes  No

**If yes**, for how long in total? ……………………………………………………..

**And**, are you currently using hormone replacement therapy?  Yes  No

**INFORMATION ABOUT BREAST CANCER DIAGNOSIS AND TREATMENT**

26. Have you ever had surgery for a benign lump or cyst in the breast?  Yes  No

27. How old were you when you were first diagnosed with breast cancer? Years

Which breast was affected?  Left Right

What type of cancer was it? (i.e invasive/non invasive) …………………………………

Which hospital was the first diagnosis made? ………………………………..

Name of doctor: ………………………………..

**INFORMATION ABOUT YOUR FAMILY**

We would like to ask you some questions about your **blood relatives**. Please only include details about your natural parents, children who are genetically related to you (not adopted children or step-children) and full brothers and sisters. We ask for this information because we are trying to understand about cancer in families. **Please answer all questions.**

28**. INFORMATION ABOUT YOUR MOTHER**

What is/was your mother’s **full** name? …………………………………………………………………

What was her maiden name? …………………………………………………………………

How would you describe her ethnicity?

Malay  Chinese  Indian  Other, please specify

Her most recent town of residence

*(or if deceased, last town and county of residence)*  …………………………………………………………………

What was her date of birth? (dd/mm/yyyy) //

Is she still alive?  Yes  No

**If no,** what was her date of death? //

Did she ever have cancer?  Yes  No

**If yes***,* what type of cancer? …………………………………………………………………

**And** how old was she when it was diagnosed?  Years

29. **INFORMATION ABOUT YOUR FATHER**

What is/was your father’s full name? …………………………………………………………………

How would you describe his ethnicity?

Malay  Chinese  Indian  Other, please specify

His most recent town of residence

*(or if deceased, last town and county of residence)* …………………………………………………………………

What was his date of birth? //

Is he still alive?  Yes  No

**If no,** what was his date of death? //

Did he ever have cancer?  Yes  No

**If yes***,* what type of cancer? …………………………………………………………………

**And** how old was he when it was diagnosed?  Years

30. **INFORMATION ABOUT YOUR BROTHERS AND SISTERS**

How many full brothers and sisters do you have/have you had?

| **Full name** | **Date of Birth**  **(day/month/yr)** | **Sex** | **Did he/she ever have cancer?** | **If yes, what type of cancer?** | **Age when diagnosed?** | **Is he/she still alive?** | **If not, what was their date of death?** | **Town of residence** |
| --- | --- | --- | --- | --- | --- | --- | --- | --- |
|  |  |  |  |  |  |  |  |  |
|  |  |  |  |  |  |  |  |  |
|  |  |  |  |  |  |  |  |  |
|  |  |  |  |  |  |  |  |  |
|  |  |  |  |  |  |  |  |  |
|  |  |  |  |  |  |  |  |  |
|  |  |  |  |  |  |  |  |  |
|  |  |  |  |  |  |  |  |  |
|  |  |  |  |  |  |  |  |  |
|  |  |  |  |  |  |  |  |  |
|  |  |  |  |  |  |  |  |  |
|  |  |  |  |  |  |  |  |  |
|  |  |  |  |  |  |  |  |  |
|  |  |  |  |  |  |  |  |  |

31. **INFORMATION ABOUT YOUR CHILDREN**

How many children do you have/have you had?

| **Full name** | **Date of Birth**  **(day/month/yr)** | **Sex** | **Did he/she ever have cancer?** | **If yes, what type of cancer?** | **Age when diagnosed?** | **Is he/she still alive?** | **If not, what was their date of death?** | **Town of residence** |
| --- | --- | --- | --- | --- | --- | --- | --- | --- |
|  |  |  |  |  |  |  |  |  |
|  |  |  |  |  |  |  |  |  |
|  |  |  |  |  |  |  |  |  |
|  |  |  |  |  |  |  |  |  |
|  |  |  |  |  |  |  |  |  |
|  |  |  |  |  |  |  |  |  |
|  |  |  |  |  |  |  |  |  |
|  |  |  |  |  |  |  |  |  |
|  |  |  |  |  |  |  |  |  |
|  |  |  |  |  |  |  |  |  |
|  |  |  |  |  |  |  |  |  |
|  |  |  |  |  |  |  |  |  |

32. **HAVE ANY OF YOUR OTHER CLOSE BLOOD RELATIVES BEEN DIAGNOSED WITH CANCER?**  Yes  No

*(e.g. aunt, uncle, grandmother, grandfather, first cousin)*

How many full **brothers** does your **mother** have/has your mother had?

How many full **sisters** does your **mother** have/has your mother had?

How many full **brothers** does your **father** have/has your father had?

How many full **sisters** does your **father** have/has your father had?

| **Relationship (please do not give their name)** | **Maternal (mother’s side)/ Paternal (father’s side)** | **What type of cancer?** | **Age when diagnosed?** | **Is he/she still alive?** | **If not, what was their age at death?** | **And, what was their year at death?** |
| --- | --- | --- | --- | --- | --- | --- |
|  |  |  |  |  |  |  |
|  |  |  |  |  |  |  |
|  |  |  |  |  |  |  |
|  |  |  |  |  |  |  |
|  |  |  |  |  |  |  |
|  |  |  |  |  |  |  |
|  |  |  |  |  |  |  |
|  |  |  |  |  |  |  |
|  |  |  |  |  |  |  |
|  |  |  |  |  |  |  |
